# The Ramazzini Institute 13-week pilot study glyphosate-based herbicides administered at human-equivalent dose to Sprague Dawley rats: effects on development and endocrine system

**DOI:** 10.1186/s12940-019-0453-y

**Published:** 2019-03-12

**Authors:** Fabiana Manservisi, Corina Lesseur, Simona Panzacchi, Daniele Mandrioli, Laura Falcioni, Luciano Bua, Marco Manservigi, Marcella Spinaci, Giovanna Galeati, Alberto Mantovani, Stefano Lorenzetti, Rossella Miglio, Anderson Martino Andrade, David Møbjerg Kristensen, Melissa J. Perry, Shanna H. Swan, Jia Chen, Fiorella Belpoggi

**Affiliations:** 1 Cesare Maltoni Cancer Research Center (CMCRC), Ramazzini Institute (RI), Via Saliceto, 3, 40010, Bentivoglio, Bologna, Italy; 2 Department of Veterinary Medical Sciences, University of Bologna, Bologna, Italy; 3 Department of Environmental Medicine and Public Health, Icahn School of Medicine at Mount Sinai, New York, USA; 4 Department of Agricultural Sciences, University of Bologna, Bologna, Italy; 5 Department of Food Safety, Nutrition and Veterinary Public Health, Istituto Superiore di Sanità, Rome, Italy; 6 Department of Statistical Sciences, University of Bologna, Bologna, Italy; 7 Department of Physiology, Division of Biological Sciences, Federal University of Paraná, Curitiba, Paraná Brazil; 8 Danish Headache Center, Department of Neurology, Rigshospitalet, University of Copenhagen, 1165 Copenhagen, Denmark; 9 Department of Environmental and Occupational Health, Milken Institute School of Public Health, The George Washington University, Washington, DC, USA

## Abstract

**Background:**

Glyphosate-based herbicides (GBHs) are broad-spectrum herbicides that act on the shikimate pathway in bacteria, fungi, and plants. The possible effects of GBHs on human health are the subject of an intense public debate for both its potential carcinogenic and non-carcinogenic effects, including potential effects on the endocrine system The present pilot study examine whether exposure to GBHs at the dose of glyphosate considered to be “safe” (the US Acceptable Daily Intake - ADI - of 1.75 mg/kg bw/day), starting from in utero life, affect the development and endocrine system across different life stages in Sprague Dawley (SD) rats.

**Methods:**

Glyphosate alone and Roundup Bioflow, a commercial brand of GBHs, were administered in drinking water at 1.75 mg/kg bw/day to F0 dams starting from the gestational day (GD) 6 (in utero) up to postnatal day (PND) 120. After weaning, offspring were randomly distributed in two cohorts: 8 M + 8F/group animals belonging to the 6-week cohort were sacrificed after puberty at PND 73 ± 2; 10 M + 10F/group animals belonging to the 13-week cohort were sacrificed at adulthood at PND 125 ± 2. Effects of glyphosate or Roundup exposure were assessed on developmental landmarks and sexual characteristics of pups.

**Results:**

In pups, anogenital distance (AGD) at PND 4 was statistically significantly increased both in Roundup-treated males and females and in glyphosate-treated males. Age at first estrous (FE) was significantly delayed in the Roundup-exposed group and serum testosterone concentration significantly increased in Roundup-treated female offspring from the 13-week cohort compared to control animals. A statistically significant increase in plasma TSH concentration was observed in glyphosate-treated males compared with control animals as well as a statistically significant decrease in DHT and increase in BDNF in Roundup-treated males. Hormonal status imbalances were more pronounced in Roundup-treated rats after prolonged exposure.

**Conclusions:**

The present pilot study demonstrate that GBHs exposure, from prenatal period to adulthood, induced endocrine effects and altered reproductive developmental parameters in male and female SD rats. In particular, it was associated with androgen-like effects, including a statistically significant increase of AGDs in both males and females, delay of FE and increased testosterone in female.

**Electronic supplementary material:**

The online version of this article (10.1186/s12940-019-0453-y) contains supplementary material, which is available to authorized users.

## Background

Glyphosate [IUPAC chemical name N-(phosphonomethyl)glycine] is the active ingredient of all glyphosate-basedherbicides (GBHs), which is the most widely applied pesticide worldwide including the commercial formulation “Roundup” [6, 31]. Since the late 1970s, the volume of GBHs applied has increased around 100-fold [31]. The widespread exposure of human population to GBHs has raised public health concerns, including potential effects on the endocrine system, for example by inhibiting aromatase enzyme activity [14, 39] and/or by activating estrogen receptors (ERs) [1, 21, 46, 49]. In vitro, the reduction in aromatase activity has been reported in placental and embryonic human cells treated with low concentrations of Roundup [5, 39] and other formulations [14]. In tumor MA-10 Leydig cells, treated with different concentrations of Roundup, the expression of aromatase and steroidogenic acute regulatory protein (StAR) also decreased [52]. GBHs and their adjuvants were able to induce proliferative effects in human hormone-dependent breast cancer cells, further suggesting an endocrine-related mode of action [29, 49]. A more recent in vitro study also showed that human sperm incubation with glyphosate at 1 mg/L reduced sperm motility possibly related to sperm mitochondrial dysfunction [3].

In vivo, sexual development is controlled by hormones and is therefore highly sensitive to exogenous substances with endocrine-related effects. In rats, different studies have investigated the effects of high doses of Roundup administered to rats prenatally and postnatally on sexual maturity. A range of significant effects were observed, including i) both increased or reduced concentration of total testosterone (TT) in males treated with Roundup formulation (Monsanto of Brazil) containing 18% (*w*/*v*) polyoxyethyleneamine (surfactant) [11, 41]; ii) increased 17β-estradiol (E2) serum concentrations in males treated with Roundup Transorb formulation [41]; iii) delayed sexual maturation in females, as indicated by delayed vaginal opening, and iv) reduced spermatogenesis [11]. Similarly, peripubertal exposure to Roundup Transorb retarded sexual maturation, increased alterations of seminiferous tubules and reduced TT in male Wistar rats even at the lowest dose level tested i.e. 5 mg/kg bw/day [42]. Finally, also an alteration in pituitary hormones was observed in adult rats exposed to Roundup [35].

Pure glyphosate might be less potent than GBHs (such as Roundup formulations) in terms of reproductive toxicity. Testicular toxicity and reduced sperm counts, but no hormone variations, were observed in sexually mature male Sprague-Dawley (SD) rats treated active ingredient glyphosate, only at the highest dose level of 500 mg/kg bw/day [10].

In addition, evaluation of glyphosate and GBHs by international agencies is not without controversies. No evidence of interaction of glyphosate with the estrogen pathway was detected in the Endocrine Disruptor Screening Program (EDSP) conducted by the US Environmental Protection Agency (EPA) [50]. However, in Fish Early Life-Stage Toxicity (Threespine Stickleback) assay, EPA dismissed statistically significant differences in plasma vitellogenin, consistent with estrogenic activity, because of a non-monotonic dose response [51]. The European Food Safety Authority (EFSA) concluded in 2017 that the weight of evidence did not support endocrine disrupting properties of GBHs through estrogen, androgen, thyroid or steroidogenesis (EATS) modes of action. However, in a prior 2015 report EFSA noted that ‘signs of endocrine activity could not be completely ruled out’ in some of these assays [51].

Because to date relatively few human health studies have been conducted, the epidemiological evidence of GBH effects on reproductive and developmental health outcomes is too limited to draw conclusions. The Ontario Farm Family Health Study (OFFHS) showed a significant association between preconception exposure to pesticide products containing glyphosate and increased risk of spontaneous abortion [4, 43]. A recent small study found a significant association between urine glyphosate concentration in pregnant women and shorter gestational length [37]. A recent human study also suggested that maternal exposure to organophosphate has been associated with a no significant dose-related elongation of anogenital distance (AGD) in the female newborns at 3 months of age [12]. In rodents and primates, AGD is 50–100% longer in males than females. The increased growth of this region occurs in response to androgens and is related to fetal androgens exposure in early development; higher in utero androgen exposure results in longer AGD in both sexes. Many epidemiological studies have reported population data on AGD and have shown links between AGD and testicular function and androgen action across a wide range of clinical outcomes [18]. Therefore, AGD has emerged as an informative and valid biomarker to assess the effects of a sub-optimal hormonal environment on human reproductive development from fetal to adult life [48].

Taken together, both in vitro and in vivo published studies to date, present conflicting findings. Glyphosate alone or GBHs exposure combined may related to adverse developmental or reproductive effects, albeit many studies used very high doses of exposure. In vivo studies have been performed primarily in male rats, from different strains, at different life stages and using different endpoints. It is also not clear whether the possible adverse effects are due to endocrine disruption of GBHs [29]. Interpretation of the available data, particularly for the measurement of circulating hormones which are known to have large variation, should also take into account that different animal models can introduce biological variability, along with no comparable study designs and pre-analytical conditions [7].

The present pilot study examined whether exposure to GBH at a dose of glyphosate considered to be “safe”, i.e. the US Acceptable Daily Intake (ADI) of 1.75 mg/kg bw/day, defined as the chronic Reference Dose (cRfD) determined by the US EPA [17], affect the development and endocrine system across different life stages in SD rats. To this purpose, we tested both the active substance and the commercial GBH formulation “Roundup Bioflow”.

## Methods

### Chemicals

Active ingredient glyphosate (Pestanal™ analytical standard, CAS number 1071–83-6, purity > 99.5%) was supplied from Sigma-Aldrich (Milan, Italy). The commercial formulation Roundup Bioflow (containing 360 g/L of glyphosate acid in the form of 480 g/l isopropylamine salts of glyphosate (41.5%), water (42.5%) and surfactant (16%; chemical name, CAS number and/or exact percentage have been withheld as a trade secret) was supplied from a local agricultural consortium (Consorzio Agrario dell’Emilia, Bologna, Italy). The original containers/bottles of glyphosate and Roundup Bioflow were stored in its original container and kept in a ventilated storage cabinet at room temperature (22 °C ± 3 °C) throughout the study. Suppliers provided purity data for each batch of glyphosate and Roundup Bioflow. The opening and the use date of the different batches of test substances were recorded in the raw data. An aliquot of each lot of the test article is maintained in the ventilated storage cabinet, until 5 years from the end of the main experiment. The solutions of glyphosate and Roundup Bioflow were prepared by the addition of appropriate volume of tap drinking water.

### Animals and experimental design

The entire animal experiment was performed following the rules by the Italian law regulating the use and treatment of animals for scientific purposes (Legislative Decree No. 26, 2014. Implementation of the directive n. 2010/63 / EU on the protection of animals used for scientific purposes. G.U. General Series, n. 61, March 14th 2014). All animal study procedures were performed at the Cesare Maltoni Cancer Research Centre/Ramazzini Institute (CMCRC/RI) (Bentivoglio, Italy). The study protocol was approved by the Ethical Committee of the Ramazzini Institute. The protocol of the experiment was also approved and formally authorized by the ad hoc commission of the Italian Ministry of Health (ministerial approval n. 710/2015-PR). The CMCRC/RI animal breeding facility was the supplier for the SD rats. Female breeder SD rats were placed individually in polycarbonate cages (42x26x18cm; Tecniplast Buguggiate, Varese, Italy) with a single unrelated male until evidence of copulation was observed. Each of 24 virgin female SD rats (17 weeks old, 270–315 g) was mated outbred with one breeder male rat of the same age and strain. Every day, the females were examined for presence of sperm. After evidence of mating, females were housed separately during gestation and delivery. Newborns were housed with their mothers until weaning. Weaned offspring were co-housed, by sex and treatment group, not more than 3 *per* each cage. Cages were identified by a card indicating: study protocol code, experimental and pedigree numbers, dosage group. A shallow layer of white fir wood shavings served as bedding (supplier: Giuseppe Bordignon, Treviso, Italy). Analysis of chemical characteristics (pH, ashes, dry weight, and specific weight) and possible contamination (metals, aflatoxin, polychlorinated biphenyls, organophosphorus and organochlorine pesticides) of the bedding was performed by CONSULAB Laboratories (Treviso, Italy). Pellet feed and tap drinking water were tested for possible glyphosate contamination as previously described [36].The cages were placed on racks, inside a single room prepared for the experiment at 22 °C ± 3 °C temperature and 50% ± 20% relative humidity. Daily checks on temperature and humidity were performed. The light was artificial and a light/dark cycle of 12 h was maintained. Stress-related husbandry factors were controlled: rats were kept together (same room, same rack, no more than 3 *per* each cage) and we did not relocate cages. Noise and handling time were minimized.

Two groups of SD rat dams and relative pups were treated with either glyphosate or Roundup Bioflow diluted in drinking water to achieve the desired glyphosate dose of 1.75 mg/kg bw/day. The F0 female breeders received the treatment through drinking water from gestation day (GD) 6 to the end of lactation, while the offspring (F1) continued to be exposed after weaning for additional 6 or 13 weeks. Glyphosate or Roundup solutions were freshly prepared on a daily basis depending on body weight and water consumption of dams or offspring, measured at scheduled time points. Preparation of drinking water solutions, quantification of glyphosate in water, and dosing adjustments are described in detailed by Panzacchi et al. [36]. During pregnancy and lactation, embryos and offspring (F1) were all retained in the litter and might receive the test compounds mainly through their dams (F0). The day birth occurred was designated as post-natal day 1 (PND 1) for pups and lactation day 1 (LD 1) for dams. After weaning, on PND 28, offspring were randomly distributed in two cohorts: 8 M + 8F/group animals belonging to the 6-week cohort were sacrificed at PND 73 ± 2, i.e. 6 weeks after weaning; 10 M + 10F/group animals belonging to the 13-week cohort were sacrificed at PND 125 ± 2, i.e. 13 weeks after weaning. After weaning, the offspring (F1) were treated through drinking water until sacrifice. Altogether, 108 SD rats (54 males and 54 females) were enrolled in the post-weaning treatment phase.

### Measurements in F0 dams and litters prior to weaning

Mean gestational length (duration of pregnancy) was calculated as the number of days from detection of a positive vaginal smear (GD 0) to birth of a litter. Pregnancy was confirmed by the occurrence of parturition. Dams’ body weights were recorded on GD 0, 3, 6 and then daily during gestation until parturition. During lactation, dams’ body weights were recorded at LD 1, 4, 7, 10, 13, 16, 19, 21 and 25 (last measurement before weaning). Pups’ body weight by sex and litter was determined on PND 1, 4, 7, 10, 13, 16, 19, 21 and 25. Dams’ feed and water consumption were recorded twice weekly during gestation (GD 0, 3, 6, 9, 12, 15, 18, 21), whereas during lactation were measured at LD 1, 4, 7, 10, 13, 16, 19, 21 and 25.

To determine the number of pups born to each dam as accurately as possible, we examined cages at frequent intervals during parturition. Dead pups were removed when found and sexed when possible. Sex was determined on PND 1 and sex ratio data was presented as ratio of males to females. The mean litter size was calculated on PND 0 (within 24 h from delivery), 1, 4, 7, 10, 13, 16, 19, 21, 25. Litter size included dead as well as live offspring. Dead pups were visually examined by floating the lungs in saline, to distinguish if they were stillborn (died in utero) or died shortly after birth. Live-birth index was calculated at PND 0 as (number of pups born alive / total number of pups born) × 100. Survival index, calculated as (total number of live pups at designated time point / number of live pups born) × 100, was measured on PND 1, 4, 7, 10, 13, 16, 19, 21 and 25. For all the pups, ano-genital distance (AGD), reflecting the linear distance between the genital tubercle and the anus, was measured on PND 4, using a Vernier caliper calibrated with a micrometer stage. Measurement was made from the caudal margin of the anus to the caudal margin of the genital tubercle [22]. Pup body weight was collected on the day the AGD was measured.

### Post weaning endpoints up to adulthood

After weaning body weight was measured twice a week, until PND 73 ± 2, then weekly until PND 125 ± 2 and before terminal sacrifices, the means of individual body weights were calculated for each group and sex. Daily water and feed consumption per cage were measured twice a week, until PND 73 ± 2, then weekly until PND 125 ± 2; the means of individual consumptions were calculated for each group and sex. Time to vaginal opening (VO) was determined by daily inspection of all female pups starting on PND 28. The criterion was met for female rats when a complete rupture of the membranous sheath covering the vaginal orifice was observed [24]. The body weight of each female was recorded on the day that this was observed. Time to balano-preputial separation (BPS) was determined by daily inspection of all males beginning on PND 35. The criterion for the day complete preputial separation was present when the prepuce was observed to completely retract from the head of the penis [24]. The body weight of each male was recorded on the day that this was observed. The female rats belonging to the developmental cohort (8F/group) were also monitored for the time to first estrous (FE), defined as the first day on which only cornified epithelial cells were observed on a vaginal smear, determined by vaginal cytology for 14 consecutive days, starting 3 days after vaginal opening was observed [32].

### Estrous cycle characterization

Starting on approximately PND 95 and for the duration of 3 weeks, daily vaginal lavage was performed on female rats belonging to the 13-week cohort (10 F/group). To reduce variability, vaginal cytology samples were collected by vaginal lavage at the same time of the day over the course of the experiment, in the mid-morning, between 10:00 and 13:00. Collection, processing and vaginal smear evaluation was performed as described by us previously [26] .

### Necropsy

All the animals were anesthetized by inhalation of a mixture of CO_2_/O_2_ (70 and 30% respectively), and sacrificed by drawing blood by cava vein. The time and date of necropsy were recorded. Five days after weaning (corresponding to 49 ± 2 days of treatment), dams were sacrificed and the following organs were collected and alcohol fixed during necropsy: mammary glands (4 sites: axillary and inguinal, right and left), adrenal glands, uterus (including cervix), ovaries, vagina. The adrenal glands, uterus and ovaries were also weighed as soon as possible after dissection. For testosterone concentration determination, blood was collected and serum removed by centrifugation and stored at − 80 °C until analysis.

All male and female pups belonging to both cohorts were sacrificed on PND 73 ± 2 and PND 125 ± 2. The following organs and tissues were collected and alcohol-fixed: mammary gland (4 sites: axillary and inguinal, right and left), thyroid and parathyroid, adrenal glands, bladder and prostate, seminal vesicle/coagulating gland, left and right testis with epididymis (half of the right testis and the whole right epididymis were frozen in liquid nitrogen and stored at − 80 °C until evaluation), uterus (including cervix), ovaries and vagina.

During necropsy, other tissues displaying anomalies and all gross lesions were collected, if present. Adrenal glands, bladder and prostate, seminal vesicle/coagulating gland, left testis, left epididymis, uterus (including cervix) and ovaries were weighed as soon as collected. In case of paired organs, both organs were preserved. The organ weight was related to body weight and was expressed as both absolute and relative organ weight.

Rats were sacrificed randomly across the 4 stages of the estrous cycle. In order to determine and allow correlation with histopathology in reproductive organs and hormone analysis, the stage of estrous cycle was determined by histological appearance of the various components of the reproductive tract for F1 females belonging to the 6-week cohort or by a vaginal smear examined on the day of necropsy for F1 females belonging to the 13-week cohort.

### Sperm analysis

Sperm analyses were performed on each male animal from both cohorts, at scheduled necropsies on PND 73 ± 2 and PND 125 ± 2.

#### Sperm counts, daily sperm production, and sperm transit time through the epididymis

At necropsy, half of the right testis and the whole right epididymis were frozen in liquid nitrogen and stored at − 80 °C until evaluation. Spermatids resistant to the process of testicular homogenization and spermatozoa present in the caput/corpus and cauda epididymis were counted as previously described by Robb et al. [40] with slide adaptations described as follows. The tunica albuginea was removed from the (half) testicle, and a sample of the parenchyma was weighed and homogenized in 5 ml saline-TritonX-100 0.05%. The samples were then diluted 10–20 times in saline, and the mature spermatids resistant to homogenization (step 17–19 spermatids) were counted using a Thoma chamber. Four fields *per* animal were recorded, and the numbers of spermatids *per* gram of testis were calculated. To calculate the daily sperm production (DSP) these values were subsequently divided by 6.1, which is the number of days step 17–19 spermatids are present in the seminiferous epithelium [40]. Similarly, the segments of the epididymis (caput, corpus and cauda) were cut with a scissor, weighed, homogenized, diluted and counted as described for the testes. The number of spermatozoa in each homogenate was determined and the total number of spermatozoa for each segment of the epididymis calculated. The epididymal sperm transit time through the epididymal caput/corpus and cauda was calculated by dividing the number of spermatozoa present in each portion of the epididymis by the DSP of the associated testis [2].

#### Sperm morphology

To assess the percentage of morphologically abnormal sperm half of left cauda epididymis of each rat was transferred to a Petri dish containing 2.5 ml (for 70 day old animals) or 3.5 ml (for 120 day old animals) of Dulbecco’s phosphate-buffered saline prewarmed to 37 °C, cut in 2–3 pieces and incubated of approximately 3 min at 37 °C, periodically gently swirling the Petri dish and its contents to facilitate release of sperm from the cauda.

Dried smears of epididymal spermatozoa were stained with 1% Eosin Y for 30 min and evaluated at 400 x magnification. Five hundred spermatozoa *per* rat were evaluated and scored as morphologically normal or abnormal according to the presence or absence of head or tail defects [8, 25].

### Histopathology

After fixation, samples were trimmed, processed, embedded in paraffin wax, sectioned to a thickness of 4–5 μm and then processed in alcohol-xylene series and stained with hematoxylin and eosin for microscopic evaluation. Histopathology evaluation was performed in blind by at least two pathologists. At least one senior pathologist peer reviewed all lesions of oncological interest as well as any lesion of dubious interpretation. In the pathological diagnosis, all the pathologists used the same evaluation criteria and the same classification based on international standard criteria (INHAND, NTP) described in the specific Standard Operating Procedures and long adopted at the CMCRC/RI. The diagnoses are reported in the experimental registries.

### Hormone analysis

Serum concentration of free (fT) and total testosterone (TT); 5α-dihydrotestosterone (DHT); 17β-estradiol (E2) and Sex Hormone Binding Globulin (SHBG) were measured in duplicates by solid phase enzyme-linked immunosorbent assays (ELISAs). Blood sera, obtained and stored as described above, were used to assess the quantitative measurements in rat serum of E2, fT, TT, DHT and SHBG by ELISA based on the principle of the competitive binding, using the following commercial kits: “Estradiol rat ELISA” (#DEV9999), manufactured by Demeditec Diagnostics GmbH (Kiel, Germany), “Rat Free Testosterone (F-TESTO) ELISA” (#CSB-E0597r), “Rat Testosterone, T ELISA” (#CSB-E05100R); “Rat dihydrotestosterone (DHT) ELISA” (#CSB-E07879r), and “Rat sex hormone-binding globulin (SHBG) ELISA” (#CSB-E12118r), manufactured by Cusabio Biotech Co. Ltd. (Houston, TX, USA).

The detection range and the Lower Limit of Detection (LLD) of each ELISA kit was 2.5–1280 pg/mL and 2.5 pg/mL for E2; 0.3–60 pg/mL and 0.15 pg/mL for fT; 0.13–25.6 ng/mL and 0.06 ng/mL for TT; 10–2000 pg/mL and 5 pg/mL for DHT; 375–6000 ng/mL and 375 ng/mL for SHBG. Each kit has been used following the manufacturer’s instructions and absorbance has been measured at 450 nm using a 96-well plate reader (Wallac 1420 VICTOR3™ Multilabel Reader, Perkin Elmer Inc., Waltham, MA, USA).

Plasma pituitary hormones were measured in duplicates using the “Rat Pituitary Magnetic Bead Panel” (CN: RPTMAG-86 K, Milliplex, St. Louis, MO), a Luminex® bead-based immunoassay, following manufacturers’ instructions. Using plasma samples from 40 pups (20 females and 20 males) randomly selected from the 6-week cohort (*N* = 48 total), seven plasma pituitary hormones were measured: adrenocorticotropic hormone (ACTH), brain-derived neurotrophic factor (BDNF), follicle stimulating hormone (FSH), growth hormone (GH), luteinizing hormone (LH), prolactin (PRL) and thyroid stimulating hormone (TSH). FSH and LH were also assessed in 40 pups (20 females and 20 males) randomly selected from the 13-week cohort (*N* = 60 total). Exploratory analyses of circulating BDNF and TSH results from the 6-week cohort showed marginal differences by exposure groups in male pups; thus we attempted to validate these results by measuring BDNF and TSH in all male pups (*N* = 30) from the 13-week cohort. Plasma TT was measured in duplicates in all dams (*N* = 24) using an ELISA kit, the “Testosterone Parameter Assay Kit” (CN: KGE010, R&D Systems, Minneapolis, MN), following manufacturers’ instructions.

### Statistical methods

Where data on a particular endpoint were collected from both sexes, analyses were conducted separately. All statistical tests were made using a significance level of α = 0.05. For continuous data including body weight, weight gain and organ weights, which are most often normally distributed, one-way ANOVA, followed by a Dunnett’s test was used to compare treatment versus control groups. For hormone data, which are usually non-normally distributed and have high inter-individual variability, a screening for outliers was made, based on a Box and Whisker Plot procedure and considering as outliers the values that were outside the box boundaries by more than 3 times the size of the box itself; in the case of hormone ratios, we have considered the same outliers of the single evaluation. Nonparametric Kruskal-Wallis’ tests, using beta approximation, were used in cases where data were not normally distributed (all hormones). Counting data, not normally distributed, were also analyzed with appropriate regression models. Where the observations were grouped (such as for litter data), fixed and mixed effect models were estimated (litter as random effect) and both reported. For biological parameters related to the body weight (such as the AGD), the statistical analyses were always performed included the body weight of each pup in the regression model. The incidence of pathological lesions, reported as the numbers of animals bearing lesions, were compared using a two-tail Fisher exact test. The statistical analysis was performed using Stata/IC 10.1 (for all regressions) and Statisti× 10 (for all the other tests); graphs were obtained using Microsoft Excel and Statisti× 10.

## Results

Results for maternal and reproductive outcome of dams are reported in Table 1. In dams, during both gestation and lactation, neither body weight nor weight gain differed between experimental groups. Similarly, we did not observe treatment effects for water or feed consumption during gestation or lactation. All the dams that cohabited with males achieved and maintained pregnancy; gestational length, litter size and sex ratio did not differ significantly between groups. Likewise, the mean live birth index was comparable between groups, although the number of dams with stillbirths was higher in the glyphosate (4/8) group compared to control (2/8). In pups, AGD at PND 4 was statistically significantly increased both in Roundup-treated males (*p* < 0.01) and females (*p* < 0.01) and in glyphosate-treated males (*p* < 0.01) (Table 2). Results were still significant after running multilevel linear regression models adjusted for body weight and litter as a random effect. Post-weaning body weights as well as water and feed consumption showed no difference in both female and male offspring (Table 2). In female offspring, age and body weight at VO was similar across treatment groups; however, age at FE was significantly delayed (*p* < 0.05) in the Roundup exposed group (Table 2). The box plots and dot plots of AGD and age at FE were added as supplementary material (Additional file 1: Figure S1 and Additional file 2: Figure S2). Female offspring in the control- and glyphosate-treated groups presented the FE within 6 days from the VO, while in the Roundup-treated group two out of ten females presented a more than doubled interval (12 and 14 days) between VO and FE. In female pups followed up to 13-weeks (*N* = 30), the percent of time spent in each stage of the estrous cycle did not differ between GBH-treated animals and controls (Table 3). In male offspring, exposure to glyphosate or Roundup did not affect BPS or sperm parameters (number of mature spermatids in the testis, daily sperm production, number and sperm transit time through caput/corpus and cauda epididymis and morphology) (Tables 2 and 4). There were no treatment-related gross lesions at necropsy in F0 and F1 reproductive organs and endocrine organs in either sex; absolute and relative organ weights are presented in Tables 5, 6 and 7.

**Table 1:** Maternal and reproductive outcome of dams exposed to glyphosate or Roundup Bioflow throughout pregnancy and lactation

Parameter	Control	Glyphosate	Roundup
Gestational index (%)^a^	100 (8/8)	100 (8/8)	100 (8/8)
Mean gestational length (day)^b^	22.9	23.0	23.0
Relative weight gain during pregnancy (%)^d, e^	33.1 ± 1.8	32.4 ± 2.2	33.2 ± 1.4
Relative weight gain during lactation (%)^d, f^	3.1 ± 0.7	2.5 ± 0.5	2.9 ± 0.8
Total pups (n) delivered at PND 0^g^	120	115	124
Litter size (n)^d, h^	15 ± 1.3	14.4 ± 1.9	15.5 ± 1.7
Sex ratio at birth (%)^d, i^	53.6 ± 16.9	43.2 ± 9.9	45.4 ± 12.6
Mean live birth index (%)^d, l^	95.9 ± 9.4	93.9 ± 6.8	96.1 ± 5.8
Dams with reported stillbirths (n)	2	4	3
Stillborn (n)^m^	5	7	5
Survival index at PND 1 (%)^n^	90.8 ± 10.6	93.0 ± 8.3	91.5 ± 9.0
Survival index at PND 21 (%)^n^	90.0 ± 10.0	91.3 ± 8.3	88.1 ± 8.0

^a^Gestational index = (number of females with live born / number of females with evidence of pregnancy) × 100

^b^Mean gestational length = mean number of days between GD 0 (day of positive evidence of mating) and day of parturition

^d^Mean ± standard deviation

^e^Relative weight gain during pregnancy = relative weight on the last day of pregnancy minus relative weight on the first day treatment in pregnancy, i.e. GD 6 (weight on GD 6 = 100%)

^f^Relative weight gain during lactation = relative weight on LD 21 minus relative weight on the first day of lactation, i.e. LD 1 (weight on LD 1 = 100%)

^g^Live and stillborn pups are considered

^h^Mean number of pups per litter at PND 0 (within 24 h from delivery)

^i^Sex ratio at birth = (no. of male offspring / no. of total offspring) × 100

^l^Live birth index = (no. of offspring born alive / no. of offspring born) × 100

^m^Stillborn = no. of pups died in utero

^n^Survival index = (no. of live offspring at designated time-point / no. of pups born) × 100

**Table 2:** Effects of glyphosate or Roundup Bioflow exposure on developmental landmarks and sexual characteristics of pups

Parameter	Control	Glyphosate	Roundup
Number of male pups at PND 1	58	46	53
Male pups weight at PND 1 (g)^a^	6.8 ± 0.5	7.1 ± 0.2	6.8 ± 0.4
Male pups weaning weight (g)^a, b^	50.4 ± 4.4	53.5 ± 6.0	51.8 ± 5.8
Male AGD (mm) at PND 4^a, c^	4.02 ± 0.49	4.26 ± 0.38^**^	4.34 ± 0.30^**°°^
Age (PND) at balano-preputial separation (BPS)	46.33 ± 1.85	46.78 ± 1.73	47.61 ± 2.77
Body weight at BPS (g)	202.50 ± 10.74	203.89 ± 16.68	207.50 ± 22.70
Number of female pups at PND 1	51	61	60
Female pups birth weight (g)^a^	6.4 ± 0.4	6.6 ± 0.4	6.5 ± 0.6
Female pups weaning weight (g)^a, b^	48.3 ± 5.1	50.4 ± 5.2	50.5 ± 5.1
Female AGD (mm) at PND 4^a, c^	1.70 ± 0.25	1.79 ± 0.21	1.86 ± 0.19^**°°^
Age (PND) at vaginal opening (VO)^a^	35.56 ± 1.72	35.39 ± 1.5	35.61 ± 1.14
Body weight at VO (g)^a^	108.33 ± 6.18	108.06 ± 7.10	109.44 ± 8.73
Age (PND) at First Estrous (FE)^a, d^	39.88 ± 1.25	40.13 ± 1.46	42.63 ± 3.25^#^
Number of days between VO and FE^a^	4.75 ± 0.71	5.13 ± 0.64	7.00 ± 3.78

^**^Statistically significant (*p* < 0.01) with multilevel linear regression adjusted for body weight

^°°^Statistically significant (*p* < 0.01) with multilevel linear regression adjusted for body weight and litter (random effect)

^#^Statistically significant (*p* < 0.05) with Kruskal-Wallis’ tests

^a^Mean ± standard deviation

^b^Weaning weight corresponds to PND 25

^c^AGD = ano-genital distance

^d^First estrous (FE) was evaluated only in females belonging to the 6 week cohort

**Table 3:** Estrous cycle characterization in female rats belonging to the 13-week cohort

Time (%) in cycle stages	N. females	Control	Glyphosate	Roundup
Time (%) in Diestrus	10	55.24 ± 11.70	51.43 ± 5.41	52.86 ± 5.24
Time (%) in Proestrus	10	20.95 ± 7.51	23.33 ± 5.24	23.86 ± 3.76
Time (%) in Estrus	10	23.33 ± 5.24	25.24 ± 3.92	23.81 ± 2.24

**Table 4:** Effects of glyphosate or Roundup Bioflow exposure on sperm parameters

Parameter	6-week cohort			13-week cohort		
	Control	Glyphosate	Roundup	Control	Glyphosate	Roundup
No. of males examined	8	8	8	10	10	10
Sperm number (× 10^6^/g testis)^a^	90.3 ± 22.0	83.7 ± 15.6	81.0 ± 12.0	109.4 ± 17.9	107.9 ± 11.3	119.6 ± 20.2
Daily sperm production (× 10^6^/g testis)^a^	14.8 ± 3.6	13.7 ± 2.6	13.3 ± 2.0	17.9 ± 2.9	17.7 ± 1.8	19.6 ± 3.3
Cauda epididimal sperm number (× 10^6^)^a^	51.2 ± 10.5	51.9 ± 10.5	50.9 ± 10.7	129.5 ± 28.5	125.9 ± 14.0	122.7 ± 11.0
Caput/corpus epididimal sperm number (× 10^6^)^a^	67.6 ± 6.1	66.5 ± 7.9	66.5 ± 10.6	97.0 ± 16.4	93.3 ± 13.0	93.2 ± 8.8
Sperm transit time through caput + corpus of epididymis (days)^a^	4.8 ± 1.3	5.0 ± 1.1	5.1 ± 1.0	5.5 ± 0.8	5.3 ± 1.0	4.8 ± 0.7
Sperm transit time through cauda of epididymis (days)^a^	3.5 ± 0.5	3.8 ± 0.8	3.9 ± 0.8	7.4 ± 1.8	7.2 ± 0.9	6.4 ± 1.1
Sperm transit time through epididymis in toto (days)^a^	8.3 ± 1.7	8.8 ± 1.5	8.9 ± 1.8	12.8 ± 2.2	12.5 ± 1.7	11.2 ± 1.7
Total abnormal sperm (%)^a^	6.3 ± 1.3	6.4 ± 1.8	6.3 ± 1.4	4.6 ± 1.4	4.3 ± 1.9	3.5 ± 1.3

^a:^Mean ± standard deviation

**Table 5:** Organ weights and testosterone level in dams

	Control	Glyphosate	Roundup
No. of dams examined	8	8	8
Body weight (g)^a^	302 ± 10	306 ± 15	317 ± 13
Adrenal glands^a, b^	0.110 ± 0.030 [0.036 ± 0.009]	0.106 ± 0.013 [0.035 ± 0.005]	0.106 ± 0.045 [0.033 ± 0.005]
Uterus^a, b^	0.867 ± 0.285 [0.286 ± 0.091]	0.772 ± 0.129 [0.253 ± 0.046]	0.994 ± 0.239 [0.298 ± 0.076]
Ovaries^a, b^	0.239 ± 0.62 [0.079 ± 0.018]	0.235 ± 0.047 0.077 ± 0.017]	0.247 ± 0.052 [0.078 ± 0.016]
TT (ng/ml)^a^	3.77 ± 0.53	4.24 ± 1.48	3.90 ± 0.56

^a^Mean ± standard deviation

^b^Absolute organ weight (g). In square brackets relative organ weight (organ weight / body weight ratio × 100)

**Table 6:** Organ weights of male offspring

	6-week cohort			13-week cohort		
	Control	Glyphosate	Roundup	Control	Glyphosate	Roundup
No. of males examined	8	8	8	10	10	10
Body weight (g)^a^	321 ± 19	326 ± 16	317 ± 23	458 ± 19	454 ± 19	449 ± 15
Adrenal glands^a, b^	0.070 ± 0.014 [0.022 ± 0.005]	0.071 ± 0.015 [0.022 ± 0.004]	0.063 ± 0.009 [0.020 ± 0.002]	0.085 ± 0.025 [0.019 ± 0.005]	0.083 ± 0.024 [0.018 ± 0.005]	0.144 ± 0.190 [0.032 ± 0.041]
Testis^a, b^	1.475 ± 0.052 [0.461 ± 0.023]	1.474 ± 0.077 [0.453 ± 0.023]	1.459 ± 0.100 [0.460 ± 0.017]	1.568 ± 0.065 [0.342 ± 0.013]	1.529 ± 0.076 [0.337 ± 0.013]	1.562 ± 0.068 [0.347 ± 0.012]
Epididymis^a, b^	0.370 ± 0.041 [0.115 ± 0.011]	0.343 ± 0.040 [0.105 ± 0.010]	0.368 ± 0.038 [0.116 ± 0.006]	0.595 ± 0.039 [0.130 ± 0.008]	0.563 ± 0.043 [0.124 ± 0.008]	0.553 ± 0.026^*^ [0.123 ± 0.006]
Bladder/Prostate^a, b^	0.563 ± 0.080 [0.176 ± 0.026]	0.561 ± 0.099 [0.173 ± 0.036]	0.506 ± 0.090 [0.159 ± 0.022]	0.967 ± 0.086 [0.211 ± 0.024]	0.897 ± 0.160 [0.197 ± 0.033]	0.906 ± 0.190 [0.202 ± 0.045]
Seminal vesicles and coagulating gland^a, b^	1.129 ± 0.129 [0.353 ± 0.045]	1.110 ± 0.291 [0.339 ± 0.083]	1.235 ± 0.135 [0.389 ± 0.029]	2.049 ± 0.418 [0.446 ± 0.083]	1.879 ± 0.298 [0.414 ± 0.063]	2.055 ± 0.404 [0.457 ± 0.090]

^*^Statistically significant with Dunnett’s test (*p* < 0.05)

^a^Mean ± standard deviation

^b:^Absolute organ weight (g). In square brackets relative organ weight (organ weight / body weight ratio *100)

**Table 7:** Organ weights (g) of female offspring

	6-week cohort			13-week cohort		
	Control	Glyphosate	Roundup	Control	Glyphosate	Roundup
No. of females examined	8	8	8	10	10	10
Body weight (g)^a^	225 ± 13	219 ± 11	223 ± 11	280 ± 20	283 ± 13	283 ± 13
Adrenal glands^a, b^	0.081 ± 0.012 [0.036 ± 0.005]	0.073 ± 0.009 [0.033 ± 0.004]	0.079 ± 0.012 [0.036 ± 0.005]	0.094 ± 0.019 [0.033 ± 0.005]	0.084 ± 0.019 [0.030 ± 0.007]	0.086 ± 0.018 [0.031 ± 0.006]
Uterus^a, b^	0.499 ± 0.114 [0.221 ± 0.059]	0.531 ± 0.162 [0.241 ± 0.069]	0.619 ± 0.221 [0.277 ± 0.099]	0.539 ± 0.082 [0.192 ± 0.025]	0.575 ± 0.137 [0.202 ± 0.047]	0.589 ± 0.111 [0.209 ± 0.043]
Ovaries^a, b^	0.181 ± 0.024 [0.081 ± 0.012]	0.169 ± 0.027 [0.077 ± 0.013]	0.172 ± 0.034 [0.077 ± 0.014]	0.192 ± 0.029 [0.068 ± 0.009]	0.182 ± 0.025 [0.064 ± 0.009]	0.186 ± 0.036 [0.065 ± 0.012]

^a^Mean ± standard deviation

^b^Absolute organ weight (g). In square brackets relative organ weight (organ weight / body weight ratio × 100)

A panel of seven pituitary plasma hormones was assessed in animals from the 6-week cohort (20 males and 20 females). Most pituitary hormones were unaffected by GBH exposure, with the exception of a statistically significant increase in plasma TSH concentration observed in glyphosate-treated males compared with control animals as well as a statistically significant increase in BDNF in Roundup-treated males compared with control animals (*p* < 0.05, Tables 8). In female offspring, none of the pituitary hormones was different between treatment groups (Table 9). In light of the results observed in the 6-week cohort, we decided to measure these two pituitary hormones also in male and female offspring from the 13-week cohort (for female only few samples were available for these further analysis). Plasma TSH concentration showed an increase, even if not statically significant (*p* = 0.056) in the glyphosate-treated males and a marked and significant increase in Roundup-treated males versus control (*p* < 0.01). Plasma TSH concentration still showed a borderline significant (*p* = 0.056) increase in the glyphosate-treated males and a marked and significant increase in Roundup-treated males versus control (*p* < 0.01).

**Table 8:** Effects of glyphosate or Roundup Bioflow exposure on hormones in males (mean ± SEM)

	6-week cohort			13-week cohort		
	Control	Glyphosate	Roundup	Control	Glyphosate	Roundup
Serum Hormones						
No. of males examined	8 (8)	8 (8)	8 (8)	10 (10)	10 (10)	10 (10)
TT (ng/ml)	1.12 ± 0.12	1.02 ± 0.28	0.84 ± 0.11^a^	8.16 ± 2.86	7.65 ± 2.86	3.76 ± 0.90
fT (pg/ml)	14.53 ± 2.37	7.45 ± 2.23^b^	13.12 ± 3.74^b^	296.70 ± 123.70^c^	724.24 ± 419.22^c^	90.40 ± 29.44
DHT (pg/ml)	761.11 ± 136.21	575.28 ± 238.24	554.29 ± 145.16^a^	15,709.0 ± 5547.20	16,711.8 ± 6724.5	1980.2 ± 664.68^c**^
SHBG (ng/ml)	861.20 ± 30.24	833.24 ± 21.15	856.78 ± 32.39	917.58 ± 16.94	906.36 ± 21.62	906.51 ± 18.89
E2 (pg/ml)	1.04 ± 0.21^a^	3.29 ± 1.85	6.19 ± 2.28^b^	3.66 ± 2.57^c^	1.08 ± 0.02^d^	6.00 ± 1.11
Plasma Hormones						
No. of males examined	7 (8)	6 (8)	7 (8)	10 (10)	10 (10)	10 (10)
FSH (ng/ml)	7.00 ± 1.38	6.43 ± 1.16	7.18 ± 0.68	2.32 ± 0.40^e^	2.18 ± 0.16^f^	2.90 ± 0.28^f^
LH (ng/ml)	3.76 ± 0.79	2.87 ± 0.63	4.41 ± 0.62	1.20 ± 0.17^e^	1.25 ± 0.24^d^	1.40 ± 0.18^f^
PRL (ng/ml)	3.83 ± 0.64	3.00 ± 0.64	4.31 ± 1.32	–	–	–
GH (ng/ml)	6.03 ± 4.32^g^	23.19 ± 21.17^h^	4.38 ± 1.94^i^	–	–	–
TSH (ng/ml)	4.23 ± 0.76	8.17 ± 1.58^*^	5.57 ± 0.31^b^	1.89 ± 0.20	2.53 ± 0.25	3.69 ± 0.42^**^
ACTH (pg/ml)	346.67 ± 35.52	255.18 ± 43.29	292.26 ± 26.22	–	–	–
BDNF (pg/ml)	99.49 ± 25.32^b^	148.85 ± 37.53	171.79 ± 14.65^*^	53.83 ± 14.77^c^	58.07 ± 13.83	45.15 ± 14.64

^*^Statistically significant (*p* < 0.05) with Kruskal-Wallis’ tests

^**^Statistically significant (*p* < 0.01) with Kruskal-Wallis’ tests

^a^7 out 8

^b^6 out 8

^c^9 out 10

^d^8 out 10

^e^6 out 10

^f^7 out 10

^g^5 out 8

^h^3 out 8

^i^4 out 8

**Table 9:** Effects of glyphosate or Roundup Bioflow exposure on hormones in female (mean ± SEM)

	6 week cohort			13-week cohort		
	Control	Glyphosate	Roundup	Control	Glyphosate	Roundup
Serum Hormones						
No. of females examined	8 (8)	8 (8)	8 (8)	10 (10)	10 (10)	10 (10)
TT (ng/ml)	0.66 ± 0.064	0.75 ± 0.12	0.68 ± 0.11^a^	0.51 ± 0.06	0.72 ± 0.10	0.72 ± 0.07^b *^
fT (pg/ml)	6.49 ± 1.00^c^	6.74 ± 1.89^d^	7.70 ± 1.35^a^	9.18 ± 2.49	12.04 ± 1.25	12.52 ± 1.76^b^
DHT (pg/ml)	294.28 ± 50.40	328.34 ± 51.93^a^	488.94 ± 114.68^a^	382.93 ± 52.14	460.09 ± 60.06	268.84 ± 45.56^b^
SHBG (ng/ml)	864.82 ± 30.24	952.75 ± 54.98	903.07 ± 29.61	968.27 ± 21.39	993.44 ± 32.79	964.81 ± 27.20
E2 (pg/ml)^g^	14.95 ± 7.24	32.24 ± 8.77	66.96 ± 25.17	18.08 ± 8.49	28.48 ± 13.71	43.91 ± 9.92
Plasma Hormones						
No. of females examined	7 (8)	7 (8)	6 (8)	7(10)	5(10)	6(10)
FSH (ng/ml) ^g^	3.95 ± 2.50	2.67 ± 1.22	3.15 ± 1.65	1.58 ± 0.51	1.73 ± 0.64	1.46 ± 0.35
LH (ng/ml)^g^	5.75 ± 3.04	4.86 ± 1.93	4.52 ± 3.38	1.83 ± 0.25	2.35 ± 1.11	2.16 ± 1.28
PRL (ng/ml)^g^	102.34 ± 164.71^c^	27.49 ± 30.23	46.49 ± 31.03	–	–	–
GH (ng/ml)	12.61 ± 13.30	3.85 ± 0.97^c^	4.16 ± 2.84	–	–	–
TSH (ng/ml)	2.70 ± 1.13	3.02 ± 2.00	3.04 ± 1.53	1.29 ± 0.69^e^	1.93 ± 0.89^f^	3.03 ± 2.22^e^
ACTH (pg/ml)	331.60 ± 89.59	314.09 ± 170.60	354.95 ± 104.96	–	–	–
BDNF (pg/ml)	245.03 ± 155.68	483.62 ± 301.02	351.33 ± 177.28	25,399 ± 155.77^e^	377.79 ± 226.30^f^	249.39 ± 14,566^e^

^*^Statistically significant (*p* < 0.05) with Kruskal-Wallis’ tests

^**^Statistically significant (*p* < 0.01) with Kruskal-Wallis’ tests

^a^7 out 8

^b^9 out 10

^c^6 out 8

^d^5 out 8

^e^4 out 10

^f^2 out 10

^g^Not statistically evaluated due to insufficient sample size after clustering on the basis of the estrous cycle

BDNF plasma concentration was unaffected in this cohort. Sex steroids were measured in all animals of both 6-week and 13-week cohorts, providing data as follows:

- TT serum concentration significantly increased in Roundup-treated female offspring from the 13-week cohort compared to control animals (*p* < 0.05); TT showed an increase in the glyphosate-treated group, even if not statistically significant (Table 9). However, serum TT concentration did not differ by GBH exposure in the younger female offspring from 6-week cohort (Table 9) or in the male offspring (Table 8).
- In males, serum DHT concentration was markedly and significantly decreased in the Roundup-treated group (13-week cohort) compared to control animals (*p* < 0.01). The box plots and dot plots of DHT was added as supplementary material (Additional file 3: Figure S3).
- No significant differences in serum fT and SHBG concentrations were observed in males or females belonging to both the cohorts.
- E2 serum concentration did not show statistically significant differences in male offspring exposed to glyphosate or Roundup in both the cohorts. In females, right before sacrifice, the endocrine status (diestrus, proestrus and estrus) for individual rats was assessed by vaginal smears. We could not statistically evaluate E2 (as well as FSH, LH and PRL concentrations) in the different female groups with reference to the stages of the estrous cycle due to insufficient sample size after clustering for endocrine status.

The data on each hormone assay coefficient of variation was provided as supplementary material (Additional file 4: Figure S4 and Additional file 5: Figure S5). Hormone ratios were calculated as indicators of the general balance between hormones (Tables 10 and 11) and, in particular, of the sex steroid hormone bioavailability.

- The ratio between TT and SHBG (e.g. TT/SHBG), currently used as an indicator of testosterone bioavailability and known as the fT index, was significantly increased (*p* < 0.05) in the Roundup-treated females (13-week cohort), but not 6-week females or in any of the males.
- The E2/SHBG ratio, an indicator of E2 bioavailability known as free estradiol index (FEI), significantly increased in Roundup-treated males belonging to the 6-week cohort (*p* < 0.05), whereas no effects were observed in E2/SHBG ratio in glyphosate-treated SD male rats.
- The fT/TT ratio significantly decreased in glyphosate- (6-week cohort) and in Roundup-treated males (13-week cohort) (*p* < 0.05) but not in any of the females.
- Both male and female Roundup-treated animals belonging to the 13-week cohort showed a marked decrease in DHT/TT ratio (*p* < 0.01). No statistically significant differences were observed in younger males and females (6-week cohort).
- No statistically significant differences were observed the E2/TT ratio in males and females.

**Table 10:** Effects of glyphosate or Roundup Bioflow exposure on hormone ratios in males (Mean ± SEM)

Hormone ratios (ng/ml)	6-week cohort			13-week cohort		
	Control	Glyphosate	Roundup	Control	Glyphosate	Roundup
No. of males examined	8 (8)	8 (8)	8 (8)	10 (10)	10 (10)	10 (10)
fT/TT (x 10^−3^)	12.5 ± 1.35	8.67 ± 0.83^a *^	10.8 ± 0.45^b^	53.0 ± 11.1^c^	85.0 ± 27.5^c^	24.7 ± 4.03^*^
DHT/TT	0.658 ± 0.081	0.511 ± 0.087	0.614 ± 0.122^d^	2.195 ± 0.37	2.490 ± 0.70	0.65 ± 0.15^c **^
E2/TT (× 10^− 3^)	1.01 ± 0.19^d^	2.65 ± 1.15	10.1 ± 5.17^d^	1.59 ± 1.23^c^	0.52 ± 0.14^e^	1.84 ± 0.42^c^
TT/SHBG (× 10^− 4^)	12.90 ± 1.35	12.23 ± 3.25	10.1 ± 1.37^d^	88.30 ± 31.86	86.59 ± 33.21	40.68 ± 9.36
E2/SHBG (× 10^− 6^)	1.24 ± 0.23^d^	3.90 ± 2.21	7.18 ± 2.66^*^	3.87 ± 2.69^c^	1.20 ± 0.003^f^	6.66 ± 1.25

^*^Statistically significant (*p* < 0.05) with Kruskal-Wallis’ tests

^**^Statistically significant (*p* < 0.01) with Kruskal-Wallis’ tests

^a^6 out 8

^b^5 out 8

^c^9 out 10

^d^7 out 8

^e^8 out 10

^f^7 out 10

**Table 11:** Effects of glyphosate or Roundup Bioflow exposure on hormone ratios in females

Hormone ratios (ng/ml)	6 week cohort			13 week cohort		
	Control	Glyphosate	Roundup	Control	Glyphosate	Roundup
No. of females examined	8 (8)	8 (8)	8 (8)	10 (10)	10 (10)	9 (10)
fT/ TT (x 10^−3^)	9.17 ± 0.48^a^	8.12 ± 0.55^b^	11.90 ± 2.37^c^	19.3 ± 5.20	19.0 ± 2.80	17.0 ± 1.66
DHT/ TT	0.428 ± 0.036	0.481 ± 0.060^c^	0.718 ± 0.173^c^	0.794 ± 0.11	0.702 ± 0.10	0.383 ± 0.07^d**^
E2/ TT^e^	0.012 ± 0.0003	0.045 ± 0.014	0.066 ± 0.028^c^	0.019 ± 0.005	0.035 ± 0.012	0.068 ± 0.015^d^
TT/SHBG (× 10^− 4^)	7.85 ± 1.02	8.00 ± 1.26	7.81 ± 1.35^c^	5.35 ± 0.62	7.27 ± 0.98	7.5 ± 0.61^*^
E2/SHBG (× 10^− 5^)^e^	1.69 ± 0.78	3.51 ± 1.00	7.28 ± 2.55	1.90 ± 0.89	2.89 ± 1.41	4.87 ± 1.10 ^d^

^*^Statistically significant (*p* < 0.05) with Kruskal-Wallis’ tests

^**^Statistically significant (*p* < 0.01) with Kruskal-Wallis’ tests

^a^6 out 8

^b^5 out 8

^c^7 out of 8

^d^9 out 10

^e^Not statistically evaluated due to insufficient sample size after clustering on the basis of the estrous cycle

## Discussion

Roundup Bioflow, when administered to SD rats from in utero through adulthood at a dose level corresponding to the glyphosate RfD defined by the US EPA (1.75 mg/kg bw/day), elicited subtle but potentially adverse effects on reproductive development and hormone concentrations. In particular, two apical endpoints were found to be statistically significantly affected:

- AGD was increased in both males (glyphosate group) and females (Roundup group);
- age at FE in females was significantly delayed (Roundup group).

Statistically significant changes in hormone profiles, indicators of hormonal activity, were also observed. In the 6 week cohort, glyphosate and Roundup elicited treatment related effects only in males (females were not affected in this shorter window of exposure), as follows:

- increased TSH and decreased fT/TT in glyphosate treated rats;
- increased BDNF and E2/SHBG in Roundup treated rats

In the 13-week cohort, only Roundup and not glyphosate induced sex steroid hormones alterations in both sexes, including:

- decreased DHT; increased TSH; decreased fT/TT in males;
- increased TT and TT/SHBG in females;
- decreased DHT/TT ratio in both sexes.

Overall, these effects indicate an impact on pre- and peri-pubertal sexual maturation. Noticeably, the pattern of effects also indicate specific sex-related and treatment-related differences. In particular, the effects of treatment with glyphosate were essentially limited to increased AGD and TSH concentration, and both changes were specific to males. Conversely, Roundup Bioflow seemed to affect both females and males, resulting in a statistically significant increased AGD and sexual hormones imbalances in both the cohorts.

Considering these outcomes with a weight of evidence approach, statistically significant differences in apical endpoints (AGD and FE) together with changes in hormonal activity detected in both the treatment groups should be should be taken into account suggesting evidence of concern for reproductive toxicity via an endocrine disruption mechanism [33]. Indeed, a longer AGD at birth in both sexes and an increased age at FE, together with the increased TT in females offspring, are considered endpoints for androgen-mediated activity by the weight of evidence assessment [33]. As already pointed out, the effects of the treatments on hormone concentrations in our study were clearly different between the two sexes. Sex-related differences in toxicological responses are frequently observed with EDCs, associated with differences in hormone regulation in the two sexes [28]. For instance, in terms of an androgenizing mode of action, females have a baseline developmental testosterone exposure lower than males [15]. A number of animal studies have shown that the female reproductive tract is susceptible to virilisation by exogenous androgens, prior to, as well as during, the in utero masculinization programming window [13, 53]. The significant increase in AGD and the delay in the appearance of the first estrous cycle observed in Roundup-treated female SD rats is consistent with the increased developmental androgenization. The first ovulation is the true endpoint of a series of morphological and functional changes at different levels of the hypothalamic–pituitary–gonadal (HPG) axis, hence, it constitutes the unequivocal sign that puberty has been achieved [20]. We did not observe any difference in the achievement of VO, assessing the pubertal onset. Our findings are consistent with already published data: female Wistar derived-rats exposed to the glyphosate formulation Magnum Super II (Agros S.R.L., Argentina) from GD 9 to weaning to up to 200 mg/kg bw/day did not show any effect on VO opening, though they observed other noticeable long-term effects, such as a reproductive impairment when mated (lower implantation sites, lower fetal weight) [30] that is not present in our results, probably because our exposure dose was much lower. Our findings on increased TT concentration in Roundup-treated females at 13-weeks are also consistent with studies indicating that intrauterine exposure to androgenizing factors may lead to higher androgen levels later in life [54]. A significant increase in the TT/SHBG ratio (fT index), an indicator of testosterone bioavailability, was also seen in females exposed to Roundup up to 13 weeks.

In males, a prolonged, albeit of low-intensity, androgenizing effect could eventually evoke a counteracting feedback response from the HPG axis. As apical endpoint, we observed an increased AGD in both the treatment groups. Few animal studies have reported an increased male AGD after chemical exposure. In utero exposure to persistent polychlorinated biphenyls (PCBs) increased AGD in male SD rats [15], and the dioxin-like coplanar congener PCB118 administered throughout early postnatal development also increased AGD in male SD rats [23]. Hormone profiling in males, revealed a decreased DHT in Roundup treatment group (13-week cohort), suggesting an effect on TT metabolism after a prolonged exposure. In particular, the lower conversion into DHT might indicate a possible reduction in 5α-reductase enzyme activity responsible of the conversion of TT in DHT. In fact, the marked decrease in DHT/TT ratio observed in both Roundup-treated males and females could suggest an overall reduction in the biotransformation of testosterone to 5α-reduced androgen and a possible imbalance of the metabolism of androgens. However, these effects were not observed in 6-week female animals. Also, it should be noted that male gonads showed normal seminiferous tubules and sperm production; this is consistent with the fact that spermatogenesis is heavily regulated by testosterone and FSH [45], both hormones unaffected in Roundup-exposed males.

Finally, we observed a significant increase in TSH in glyphosate-treated males (6-week cohort) and Roundup-treated males (13-week cohort). Due to resource limitation, we could not investigate T3 and T4 yet, we are planning this work in the near future. We did not observe histological changes in the thyroid gland, therefore the altered concentration of TSH together with a normal histological pattern of the thyroid gland are not indicative of a thyroid-related activity. Nevertheless, our findings prompt further detailed investigation on the effect of GBH on thyroid function and development.

BDNF is a neurotrophin playing a fundamental role in survival and differentiation of selected neuronal populations during development, and in the maintenance and plasticity of neuronal networks during adulthood [44]. Our results showed a statistically significant increase in BDNF in Roundup-treated males belonging to 6-week cohort, which was not observed in older animals. BDNF is an explorative and new endpoint for neurodevelopment and the utility of neurotrophins as potential biomarkers is not completely understood. At the moment, any adverse impact of GBH exposure on neurodevelopment can only be pointed out as a topic for further investigation.

The present study has some limitations. First, this is a pilot study performed on a limited number of animals where only one dose was used. However, the dose was selected specifically for its relevance to human health risk assessment, as it is the chronic current RfD defined by the USEPA, 1.75 mg/kg bw/day and therefore a dose level expected to be “safe”. Several hormones were measured in the dams and offspring, but not all hormones were measured in all the animals, due to insufficient material for a complete data set of hormone profiling after the full-scale hematology and clinical biochemistry (data not yet published at the time when this work is presenting). Furthermore, the number and timing of blood sample collection was limited to the final sacrifice of animals, considering that this was a pilot study and that in vivo blood sampling could lead to maternal and pups stress. Another source of uncertainty, which is currently difficult to assess, is the timing of blood sampling during the necropsy session (9.00 am – 3.00 pm); a circadian-dependent modulation of circulating hormones cannot be completely ruled out. Standard errors in different hormone concentrations were wide, in relation to the relatively small group sizes and the physiological variability of hormone concentrations. In females, the estrous cycle status at the time of necropsy is another important source of variability when analyzing sexual hormone profiles. However, even if sacrificing animals on a specific day of the cycle might improve the ability to observe changes in the baseline hormone concentrations, the issue of sacrificing animals in the same cycling period (e.g estrous) is still controversial. The updated OECD Test Guidelines on reproductive-developmental toxicity do not require the sacrifice of females in the same stage of estrous, only the examination of estrous cycle on the day of necropsy is recommend to allow correlation with histopathology in reproductive organs [34]. Finally, we could not study separately the adjuvant(s) present in Roundup Bioflow (corresponding to 16% of the formulation) since the exact ingredients formulation is a trade secret. These are supposed to be surfactants, diluents or adjuvants stabilizing glyphosate and allowing its penetration in plants. It is noteworthy that the commercial formulation used in this study, Roundup Bioflow, was the representative formulated product recently evaluated for the renewal of the approval of glyphosate in EU and considered in the European Food Safety Authority peer review (MON 52276) [16]. At the same time, we covered specific windows of susceptibility relevant for the potential androgenic effects of GBHs exposure, for example in utero life and pre-puberty. Indeed, the pre-natal and post-natal development, through to puberty, presents different susceptibility windows to EDC modes of action developing organisms with different and changing susceptibility as compared to adulthood [27, 47].

The majority of significant changes observed in hormonal status emerged in the 13-week cohort (animals sacrificed at adulthood) compared to animals in the 6-week cohort (sacrificed after puberty) suggesting that more prolonged exposures were more effective in producing imbalances in the hormone concentrations. We have previously reported a possible enhanced retention of GBHs with an increasing pattern of glyphosate excreted in urine in relation to the duration of treatment in these same animals [36]. Finally, in our experimental design, the commercial formulation Roundup Bioflow was definitely more potent than glyphosate alone. Our results confirm previous observations that formulations might have stronger effects than glyphosate alone on endocrine and developmental parameters [9, 14, 19, 38]. Our results corroborate prior mixture studies [14], indicating that technical glyphosate and components of formulations may have cumulative (e.g., additive or synergistic) effects on endocrine-sensitive endpoints. Therefore, ADI calculations and other regulatory experiments should be performed not only on glyphosate, but also on its formulations and their components (that are often undisclosed)..

## Conclusions

The present study demonstrates that Roundup Bioflow exposure, at a dose level considered as “safe” (1.75 mg/kg bw/day), from prenatal period to adulthood, induced endocrine effects and altered reproductive developmental parameters in male and female SD rats. Roundup Bioflow exposure was associated with androgen-like effects, in particular in females, including a statistically significant increase of AGDs in both males and females, delay of FE and increased testosterone in females. Roundup Bioflow exposure was also associated with altered testosterone metabolism in both males and females, where a statistically significant decrease in DHT/TT ratio was observed in the longest treated group (13-week). Overall, the Roundup Bioflow elicited more and more pronounced effects than the active ingredient itself, which only increased AGD and TSH concentration in male rats in the peripubertal window (6-week cohort). However, considering that retention of any GBH in the body may increase with prolonged exposures, a life-course study on GBHs encompassing intrauterine life through to advanced adulthood is needed to confirm and further explore the initial evidence of endocrine-related effects and developmental alterations emerged in this pilot study.

## Additional files

Additional file 1: **Figure S1.** AGD index and AGD (Mean per litter) box plot (A) and dot plot (B). (DOCX 83 kb)

Additional file 2: **Figure S2.** First estrous box plot (A) and dot plot (B). (DOCX 33 kb)

Additional file 3: **Figure S3.** Male and female DHT box plot (A) and dot blot (B). (DOCX 86 kb)

Additional file 4: **Figure S4.** Effects of glyphosate or Roundup Bioflow exposure on hormones in males (mean ± SEM); coefficient of variation in square brackets. (DOCX 23 kb)

Additional file 5: **Figure S5.** Effects of glyphosate or Roundup Bioflow exposure on hormones in females (mean ± SEM); coefficient of variation in square brackets. (DOCX 23 kb)

## Abbreviations

ACTH: Adrenocorticotropic hormone
AGD: Anogenital distance
BPS: Balano preputial separation
CMCRC: Cesare Maltoni Cancer Research Center
cRfD: Chronic Reference Dose
DHT: 5α-dihydrotestosterone
DLS: Daily sperm production
EDSP: Endocrine Disruptor Screening Program
EFSA: European Food safety Authority
ELISA: Enzyme-linked immunosorbent assays
EPA: Environmental Protection Agency
ERs: Estrogen receptors
FE: First estrous
FSH: Follicle stimulating hormone
fT: Free testosterone
GBH: Glyphosate-based herbicides
GD: Gestational day
GH: Growth hormone
LD: Lactating day
LH: Luteinizing hormone
LLD: Lower Limit of Detection
PND: Post Natal Day
PRL: Prolactin
RI: Ramazzini Institute
SD: Sprague-Dawley
SHBG: Sex Hormone Binding Globulin
StAR: Steroidogenic acute regulatory protein
TSH: Thyroid stimulating hormone
TT: Testosterone
US ADI: United States Acceptable Daily Intake
VO: Vaginal opening
