# Supplementary material for: The Ramazzini Institute 13-week pilot study glyphosate-based herbicides administered at human-equivalent dose to Sprague Dawley rats: effects on development and endocrine system
Source: Environ Health. 2019 Mar 12;18:15. doi: 10.1186/s12940-019-0453-y (PMC6413565; doi:10.1186/s12940-019-0453-y)
Supplement: Supplementary file 1 — Figure S1. AGD index and AGD (Mean per litter) box plot (A) and dot plot (B). (DOCX 83 kb) [file 12940_2019_453_MOESM1_ESM.docx]

**Figure S1.** AGD index and AGD (Mean per litter) box plot (A) and dot plot (B)

| **AGD INDEX FEMALE BOX PLOT**  **A**   | **AGD INDEX MALE BOX PLOT**   |
| --- | --- |
| **AGD FEMALE BOX PLOT**   | **AGD MALE BOX PLOT**   |

Group I: Control; II: Glyphosate; III: Roundup

| **AGD INDEX FEMALE DOT PLOT**  **B**  **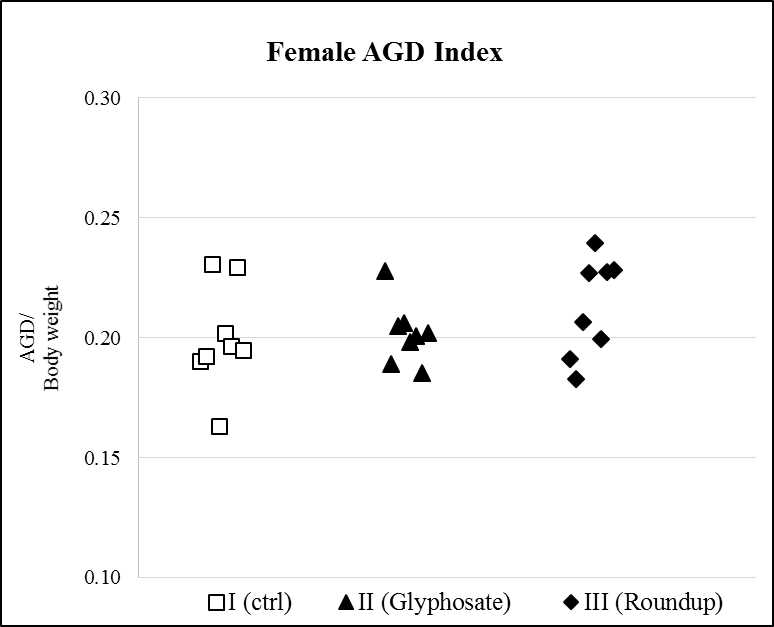** | **AGD INDEX MALE DOT PLOT**  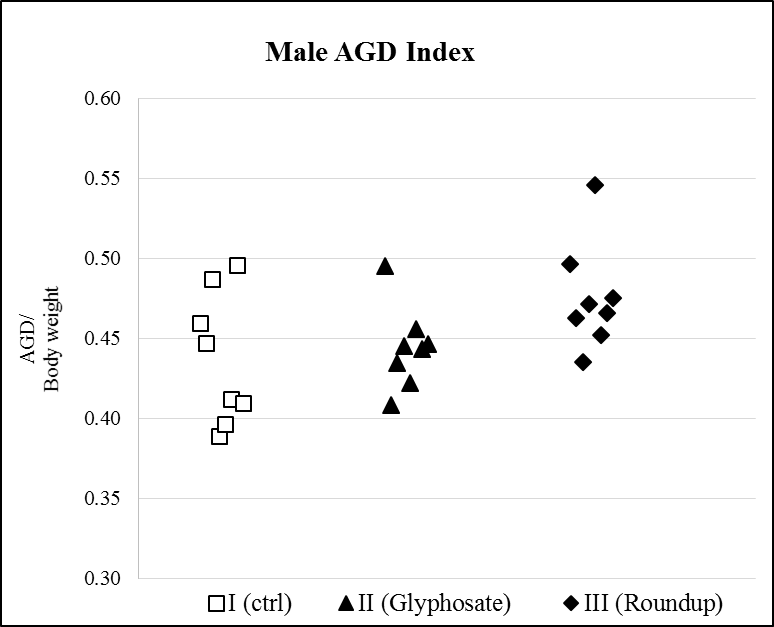 |
| --- | --- |
| **AGD FEMALE DOT PLOT**  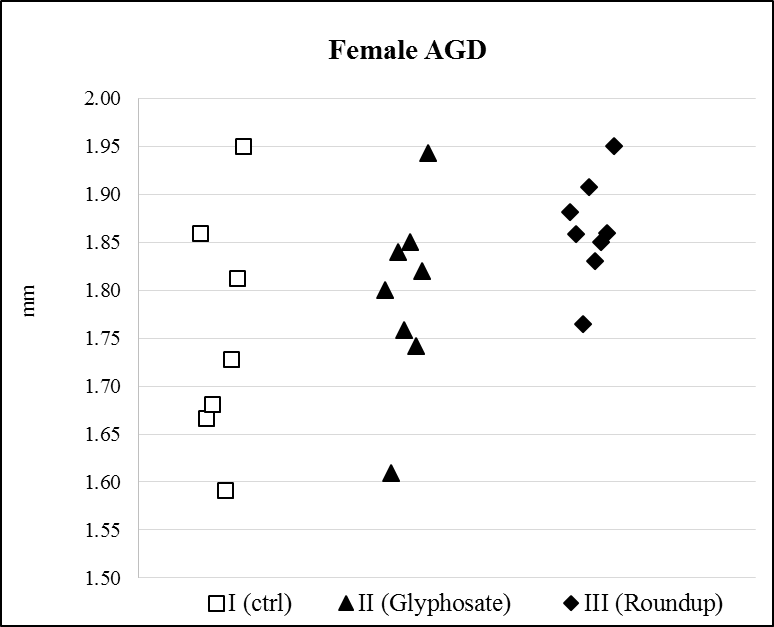 | **AGD MALE DOT PLOT**  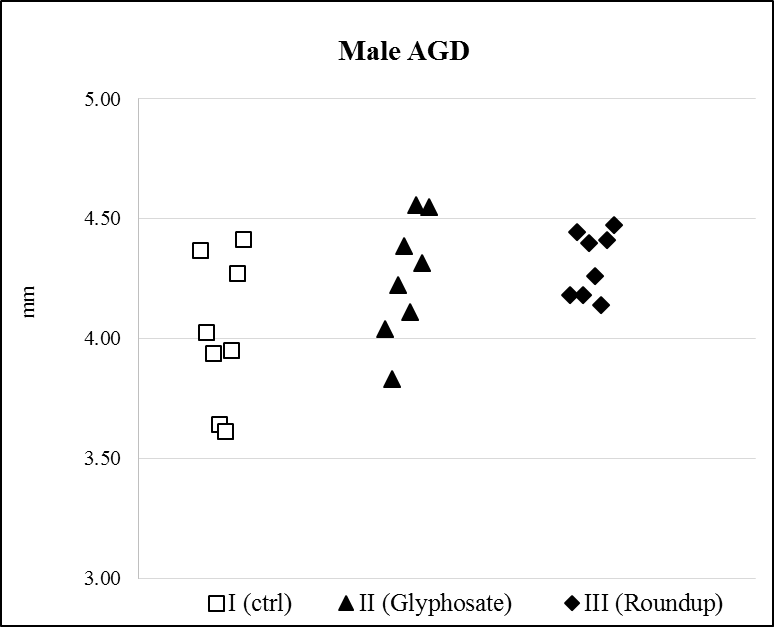 |
