# Supplementary figures and images for: The Ramazzini Institute 13-week pilot study glyphosate-based herbicides administered at human-equivalent dose to Sprague Dawley rats: effects on development and endocrine system
Source: Environ Health. 2019 Mar 12;18:15. doi: 10.1186/s12940-019-0453-y (PMC6413565; doi:10.1186/s12940-019-0453-y)

**Figure S2.** First estrous box plot (A) and dot plot (B)

| **A** | **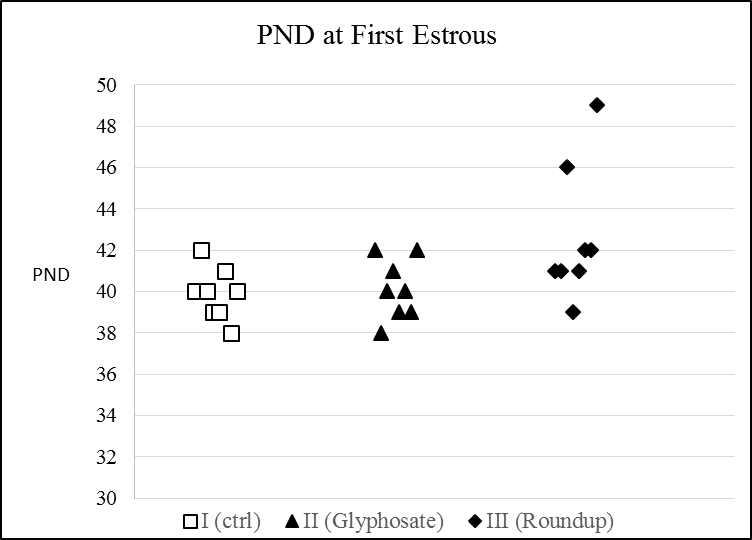**  **B** |
| --- | --- |

Group I: Control; II: Glyphosate; III: Roundup

Supplement: Supplementary file 2 — Figure S2. First estrous box plot (A) and dot plot (B). (DOCX 33 kb) [file 12940_2019_453_MOESM2_ESM.docx]
