# Supplementary material for: The Ramazzini Institute 13-week pilot study glyphosate-based herbicides administered at human-equivalent dose to Sprague Dawley rats: effects on development and endocrine system
Source: Environ Health. 2019 Mar 12;18:15. doi: 10.1186/s12940-019-0453-y (PMC6413565; doi:10.1186/s12940-019-0453-y)
Supplement: Supplementary file 3 — Figure S3. Male and female DHT box plot (A) and dot blot (B). (DOCX 86 kb) [file 12940_2019_453_MOESM3_ESM.docx]

**Figure S3. Male and female DHT box plot (A) and dot blot (B)**

Extreme values are displayed as stars (*) for possible outliers and circles (°) for probable outliers. Possible outliers are values that are outside the box boundaries by more than 1½ times the size of the box. Probable outliers are values that are outside the box boundaries by more than 3 times the size of the box.

Statistical analysis was performed also omitting extreme values, but the significance remained univariate.

| **A**  **Male Developmental cohort**   | **Male Toxicity cohort**   |
| --- | --- |
| **Female Developmental cohort**   | **Female Toxicity cohort**   |

Group I: Control; II: Glyphosate; III: Roundup

| **B**  **Male Developmental cohort**  **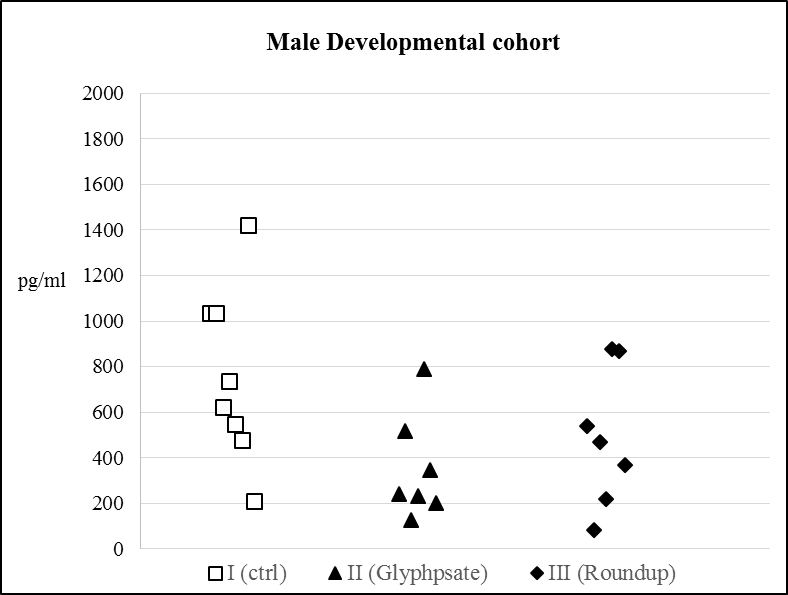** | **Male Toxicity cohort**  **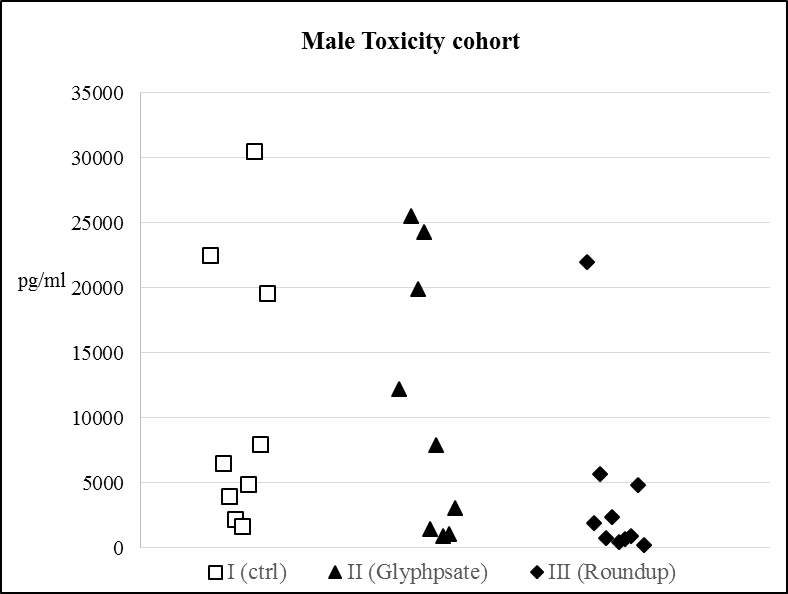** |
| --- | --- |
| **Female Developmental cohort**  **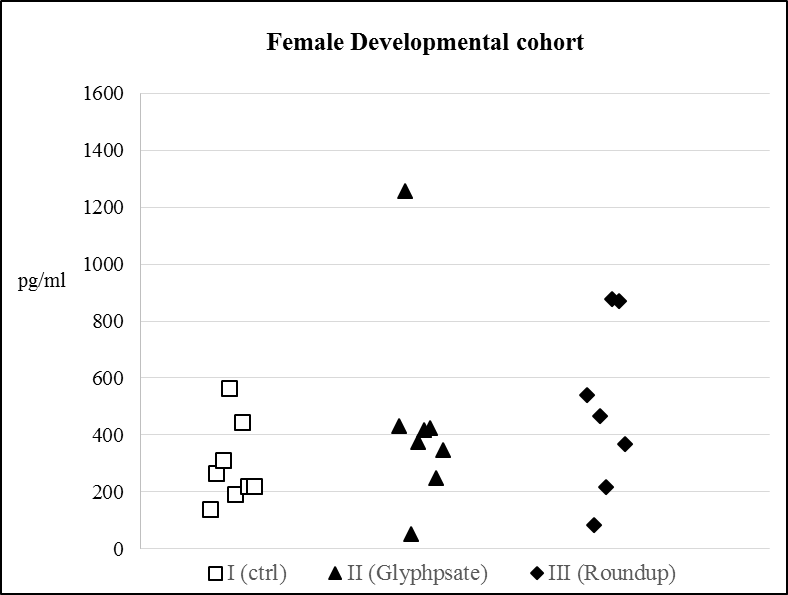** | **Female Toxicity cohort**  **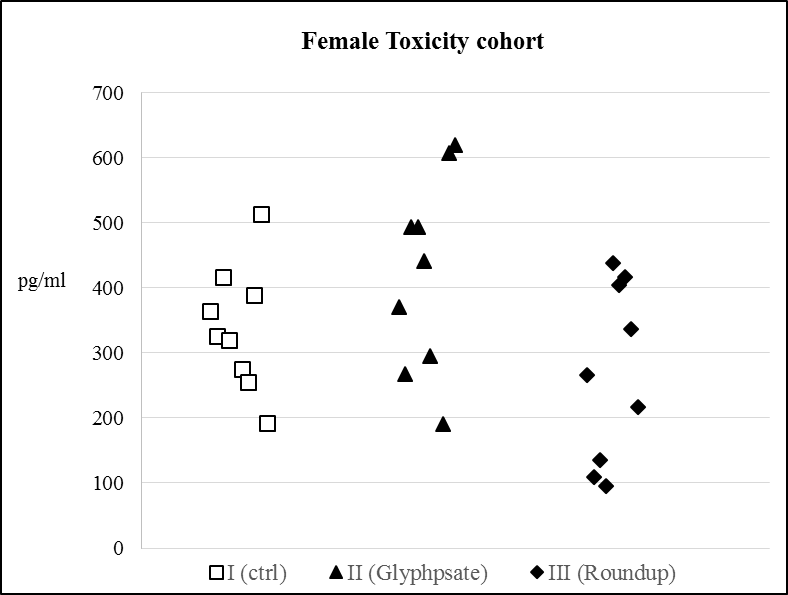** |
