# Supplementary material for: The Ramazzini Institute 13-week pilot study glyphosate-based herbicides administered at human-equivalent dose to Sprague Dawley rats: effects on development and endocrine system
Source: Environ Health. 2019 Mar 12;18:15. doi: 10.1186/s12940-019-0453-y (PMC6413565; doi:10.1186/s12940-019-0453-y)
Supplement: Supplementary file 4 — Figure S4. Effects of glyphosate or Roundup Bioflow exposure on hormones in males (mean ± SEM); coefficient of variation in square brackets. (DOCX 23 kb) [file 12940_2019_453_MOESM4_ESM.docx]

| **Serum Hormones** | **6-week cohort** | | |  | **13-week cohort** | | |
| --- | --- | --- | --- | --- | --- | --- | --- |
|  | **Control** | **Glyphosate** | **Roundup** |  | **Control** | **Glyphosate** | **Roundup** |
| No. of males examined | 8 (8) | 8 (8) | 8 (8) |  | 10 (10) | 10 (10) | 10 (10) |
| TT (ng/ml) | 1.12 ± 0.12  [0.313] | 1.02 ± 0.28  [0.764] | 0.84 ± 0.11 ^a^  [0.332] |  | 8.16 ± 2.86  [1.107] | 7.65 ± 2.86  [1.184] | 3.76 ± 0.90  [0.754] |
| fT (pg/ml) | 14.53 ± 2.37 [0.462] | 7.45 ± 2.23 ^b^  [0.734] | 13.12 ± 3.74 ^b^  [0.698] |  | 296.70 ± 123.70 ^c^  [1.251] | 724.24 ± 419.22 ^c^  [1.737] | 90.40 ± 29.44  [1.030] |
| DHT (pg/ml) | 761.11 ± 136.21  [0.506] | 575.28 ± 238.24  [1.171] | 554.29 ± 145.16 ^a^  [0.692] |  | 15709.0 ± 5547.20  [1.117] | 16711.8 ± 6724.5  [1.235] | **1980.2 ± 664.68^c^****  [1.007] |
| SHBG (ng/ml) | 861.20 ± 30.24  [0.099] | 833.24 ± 21.15  [0.072] | 856.78 ± 32.39  [0.107] |  | 917.58 ± 16.94  [0.058] | 906.36 ± 21.62  [0.075] | 906.51 ± 18.89  [0.066] |
| E2 (pg/ml) | 1.04 ± 0.21^a^  [0.531] | 3.29 ± 1.85  [1.593] | 6.19 ± 2.28 ^b^  [1.042] |  | 3.66 ± 2.57 ^c^  [2.111] | 1.08 ± 0.02^d^  [0.047] | 6.00 ± 1.11  [0.584] |
| **Plasma Hormones** | **6-week cohort** | | |  | **13-week cohort** | | |
|  | **Control** | **Glyphosate** | **Roundup** |  | **Control** | **Glyphosate** | **Roundup** |
| No. of males examined | 7 (8) | 6 (8) | 7 (8) |  | 10 (10) | 10 (10) | 10 (10) |
| FSH (ng/ml) | 7.00 ± 1.38  [0.521] | 6.43 ± 1.16  [0.440] | 7.18 ± 0.68  [0.246] |  | 2.32 ± 0.40 ^e^  [0.420] | 2.18 ± 0.16 ^f^  [0.567] | 2.90± 0.28 ^f^  [0.254] |
| LH (ng/ml) | 3.76 ± 0.79  [0.557] | 2.87 ± 0.63  [0.538] | 4.41 ± 0.62  [0.369] |  | 1.20 ± 0.17 ^e^  [1.030] | 1.25 ± 0.24 ^d^  [0.282] | 1.40 ± 0.18 ^f^  [0.338] |
| PRL (ng/ml) | 3.83 ± 0.64  [0.439] | 3.00 ± 0.64  [0.522] | 4.31 ± 1.32  [0.810] |  | - | - | - |
| GH (ng/ml) | 6.03 ± 4.32 ^g^  [1.602] | 23.19 ± 21.17 ^h^  [1.581] | 4.38 ± 1.94 ^i^  [0.886] |  | - | - | - |
| TSH (ng/ml) | 4.23 ± 0.76  [0.477] | **8.17 ± 1.58***  [0.473] | 5.57 ± 0.31 ^b^  [0.612] |  | 1.89 ± 0.20  [0.334] | 2.53 ± 0.25  [0.316] | **3.69 ± 0.42 ^**^**  [0.359] |
| ACTH (pg/ml) | 346.67 ± 35.52  [0.271] | 255.18 ± 43.29  [0.416] | 292.26 ± 26.22  [0.237] |  | - | - | - |
| BDNF (pg/ml) | 99.49 ± 25.32 ^b^  [0.623] | 148.85 ± 37.53  [0.618] | **171.79 ± 14.65***  [0.226] |  | 53.83 ± 14.77 ^c^  [0.823] | 58.07 ± 13.83  [0.753] | 45.15 ± 14.64  [1.023] |

**Figure S4. Effects of glyphosate or Roundup Bioflow exposure on hormones in males (mean ± SEM); coefficient of variation in square brackets.**

^a^: 7 out 8; ^b^: 6 out 8; ^c^: 9 out 10; ^d^: 8 out 10; ^e^: 6 out 10; ^f^: 7 out 10; ^g^: 5 out 8; ^h^: 3 out 8; ^i^: 4 out 8

* Statistically significant (p < 0.05) with Kruskal-Wallis’ tests

** Statistically significant (p < 0.01) with Kruskal-Wallis’ tests
