# Supplementary material for: The Ramazzini Institute 13-week pilot study glyphosate-based herbicides administered at human-equivalent dose to Sprague Dawley rats: effects on development and endocrine system
Source: Environ Health. 2019 Mar 12;18:15. doi: 10.1186/s12940-019-0453-y (PMC6413565; doi:10.1186/s12940-019-0453-y)
Supplement: Supplementary file 5 — Figure S5. Effects of glyphosate or Roundup Bioflow exposure on hormones in females (mean ± SEM); coefficient of variation in square brackets. (DOCX 23 kb) [file 12940_2019_453_MOESM5_ESM.docx]

| **Serum Hormones** | **6-week cohort** | | |  | **13-week cohort** | | |
| --- | --- | --- | --- | --- | --- | --- | --- |
|  | **Control** | **Glyphosate** | **Roundup** |  | **Control** | **Glyphosate** | **Roundup** |
| No. of females examined | 8 (8) | 8 (8) | 8 (8) |  | 10 (10) | 10 (10) | 10 (10) |
| TT (ng/ml) | 0.66 ± 0.064  [0.275] | 0.75 ± 0.12  [0.441] | 0.68 ± 0.11 **^a^**  [0.432] |  | 0.51 ± 0.06  [0.344] | 0.72 ± 0.10  [0.460] | **0.72 ± 0.07 ^b^***  [0.275] |
| fT (pg/ml) | 6.49 ± 1.00 ^c^  [0.380] | 6.74 ± 1.89 ^d^  [0.627] | 7.70 ± 1.35 **^a^**  [0.465] |  | 9.18 ± 2.49  [0.857] | 12.04 ± 1.25  [0.327] | 12.52 ± 1.76 ^b^  [0.422] |
| DHT (pg/ml) | 294.28 ± 50.40  [0.484] | 328.34 ± 51.93 ^a^  [0.418] | 488.94 ± 114.68^a^  [0.620] |  | 382.93 ± 52.14  [0.430] | 460.09 ±60.06  [0.413] | 268.84 ± 45.56 **^b^**  [0.508] |
| SHBG (ng/ml) | 864.82 ± 30.24  [0.099] | 952.75 ± 54.98  [0.163] | 903.07 ± 29.61  [0.093 |  | 968.27 ± 21.39  [0.070] | 993.44 ± 32.79  [0.104] | 964.81 ± 27.20  [0.089] |
| E2 (pg/ml)^g^ | 14.95 ± 7.24  [1.369] | 32.24 ± 8.77  [0.769] | 66.96 ± 25.17  [1.063] |  | 18.08 ± 8.49  [1.486] | 28.48 ± 13.71  [1.521] | 43.91 ± 9.92  [0.715] |
| **Plasma Hormones** | **6-week cohort** | | |  | **13-week cohort** | | |
|  | **Control** | **Glyphosate** | **Roundup** |  | **Control** | **Glyphosate** | **Roundup** |
| No. of females examined | 7 (8) | 7 (8) | 6 (8) |  | 7(10) | 5(10) | 6(10) |
| FSH (ng/ml) ^g^ | 3.95 ± 2.50  [0.632] | 2.67 ± 1.22  [0.455] | 3.15 ± 1.65  [0.525] |  | 1.58 ± 0.51  [0.348] | 1.73 ± 0.64  [0.370] | 1.46 ± 0.35  [0.241] |
| LH (ng/ml) ^g^ | 5.75 ± 3.04  [0.529] | 4.86 ± 1.93  [0.397] | 4.52 ± 3.38  [0.748] |  | 1.83 ± 0.25  [0.137] | 2.35 ± 1.11  [0.466] | 2.16 ± 1.28  [0.594] |
| PRL (ng/ml) ^g^ | 102.34 ± 164.71^c^  [1.609] | 27.49 ± 30.23  [1.100] | 46.49 ± 31.03  [0.667] |  | - | - | - |
| GH (ng/ml) | 12.61 ± 13.30  [1.054] | 3.85 ± 0.97 ^c^  [0.620] | 4.16 ± 2.84  [0.683] |  | - | - | - |
| TSH (ng/ml) | 2.70 ± 1.13  [0.419] | 3.02 ± 2.00  [0.662] | 3.04 ± 1.53  [0.504] |  | 1.29 ± 0.69^e^  [0.533] | 1.93 ± 0.89 ^f^  [0.463] | 3.03 ± 2.22 ^e^  [0.732] |
| ACTH (pg/ml) | 331.60 ± 89.59  [0.270] | 314.09 ± 170.60  [0.543] | 354.95 ± 104.96  [0.296] |  | - | - | - |
| BDNF (pg/ml) | 245.03 ± 155.68  [0.635] | 483.62 ± 301.02  [0.622] | 351.33 ± 177.28  [0.505] |  | 253.99 ± 155.77 ^e^  [0.613] | 377.79 ± 226.30 ^f^  [0.599] | 249.39 ± 14566 ^e^  [0.584] |

**Figure S5. Effects of glyphosate or Roundup Bioflow exposure on hormones in females (mean ± SEM); coefficient of variation in square brackets**

^a^: 7 out 8; ^b^: 9 out 10; ^c^: 6 out 8; ^d^: 5 out 8; ^e^: 4 out 10; ^f^: 2 out 10

^g^ : Not statistically evaluated due to insufficient sample size after clustering on the basis of the estrous cycle

* Statistically significant (p < 0.05) with Kruskal-Wallis’ tests

** Statistically significant (p < 0.01) with Kruskal-Wallis’ tests
